# Pre-AttentiveGaze: gaze-based authentication dataset with momentary visual interactions

**DOI:** 10.1038/s41597-025-04538-3

**Published:** 2025-02-13

**Authors:** Junryeol Jeon, Yeo-Gyeong Noh, JooYeong Kim, Jin-Hyuk Hong

**Affiliations:** 1 Gwangju Institute of Science of Technology, School of Integrated Technology, Gwangju, 61005 Republic of Korea; 2 Gwangju Institute of Science of Technology, Artificial Intelligence Graduate School, Gwangju, 61005 Republic of Korea

**Keywords:** Electrical and electronic engineering, Research data

## Abstract

This manuscript presents a Pre-AttentiveGaze dataset. One of the defining characteristics of gaze-based authentication is the necessity for a rapid response. In this study, we constructed a dataset for identifying individuals through eye movements by inducing “pre-attentive processing” in response to a given gaze stimulus in a very short time. A total of 76,840 eye movement samples were collected from 34 participants across five sessions. From the dataset, we extracted the gaze features proposed in previous studies, pre-processed them, and validated the dataset by applying machine learning models. This study demonstrates the efficacy of the dataset and illustrates its potential for use in gaze-based authentication of visual stimuli that elicit pre-attentive processing.

## Background & Summary

Human eye movements are considered as a rich modality, replete with intricate and detailed information of human internal states. It has been well established in the cognitive science field that our gaze provides not only explicit information that we are voluntarily looking somewhere but also implicit information that reveals our involuntary cues about internal state, such as emotional arousal^1^ or cognitive load^2^. Recently, methods using machine learning (ML) have attracted increasing interest for understanding eye movement data to gain insights into the states of users. Some studies have exploited gaze features–extracted from eye movement data, including fixations, saccades, and pupil diameter–to differentiate factors, such as age^3,4^, gender^5,6^, cognitive and neurological differences (including ADHD^7^, autism^8^, dyslexia^9^, Alzheimer’s disease^10^, fundus disease^11^, schizophrenia^12^, decision processes^13^, skill assessment^14^, and personality traits^15,16^). Another intriguing research direction is the exploration of unique eye movement patterns for individual identification, a concept termed “implicit gaze-based biometric authentication^17^.” This approach capitalizes on the distinct, almost inimitable nature of an individual’s gaze. Compared to other conventional authentication techniques, which may be vulnerable when unauthorized users obtain information about a password, authentication based on individual gaze traits offers a great potential for enhanced security owing to its inimitability^18^.

However, despite the potential of implicit gaze-based biometric authentication as a security measure, there are some significant challenges that should be addressed to be more applicable. These challenges stem from applying implicit biometric authentication mechanisms to gaze data. Its process encompasses an enrollment phase—where the user’s biometric information is stored as a template—and a recognition phase, where the provided biometric data is matched against the stored templates^17^. For the effective integration of gaze as biometric data, sufficient gaze information must be amassed to characterize persons, and subsequent classification must be sufficiently accurate for reliable authentication. However, the challenges associated with gaze-based authentication have not been comprehensively addressed yet, hindering its practical implementation. Moreover, further research is required to reduce the difficulties of the enrollment phase, optimize the organization of visual stimuli during gaze collection, and enhance classification performance. Previous studies have reported a wide range of the required duration of gaze interaction for authentication, from 10 seconds to up to 25 minutes^19–23^. However, to achieve comparable performance to other methods like fingerprinting, a shorter gaze duration of a few seconds, while simultaneously maintaining an accuracy, is crucial for the practical applications of gaze identification^17,24,25^. Therefore, this study focuses on the challenges associated with gaze duration.

## Visual stimuli in implicit gaze-based biometric authentication

There are two primary categories of visual stimuli for the implicit authentication: the continuous and controlled types^17^. The continuous type is relatively unobtrusive; it recognizes and authenticates unique patterns in a user’s gaze as they engage in regular activities, such as reading emails or browsing the web^26,27^. This process can be integrated seamlessly into daily tasks, making users largely unaware of its execution. In contrast, the controlled type takes a more direct approach. In this method, users are explicitly presented with certain visual stimuli, and their gaze movements in response to these stimuli are analyzed for authentication. Given the deliberate presentation of these stimuli, users are fully conscious of the authentication process as it unfolds. The controlled type can be further subdivided into static and dynamic stimuli. Static stimuli encompass non-moving visuals, such as some specific texts^19,28,29^ or stationary images^30,31^, whereas dynamic stimuli involve visual tasks related to motion, such as tracking a moving object^24,25,29^ or watching a video^29,32^.

Previous research has reported intriguing findings regarding the relationship between visual stimuli and authentication accuracy. For example, the performance accuracy of static stimuli has been observed to be intricately related to their complexity^25,33^, whereas dynamic visual stimuli, entail goal-oriented tasks such as visual search and enable quicker interactions, often yielding superior user identification accuracy^17,24,25^. Nevertheless, this advantage is not without its caveats. For example, the inherent limitation of the human eye, in which a reaction time of 100–200 ms is required to discern changes^22,34^, significantly constrains the further application of this approach. Thus, if too complex dynamic stimuli are presented within a constrained time frame, the eye’s adaptability might be limited within the short duration, rendering the process ineffective. These indicate that: 1) The intricacy of a stimulus correlates with enhanced performance. 2) Delineated tasks, such as a visual search, tend to bolster accuracy. 3) When aiming for presentations at elevated speeds, static visual stimuli are the optimal choice. Guided by these principles, we aimed to develop a distinct task which requires participants to undertake a visual search within a multifaceted static image, within a limited time.

## Evidence of very short-time gaze interaction: pre-attentive processing

To enable fast visual search, the gaze response to given visual stimuli must be rapid. To elicit fast responses to visual stimuli, we employed the stimulus construction method used in studies that utilize pre-attentive processing. Pre-attentive processing is a gaze process in which an element with a certain low-level visual feature is discovered after a very short period of fixation^35^. In this context, low-level visual features refer to primitive components of images^36^, such as colors and shapes, that combine to form a visual element of the stimuli. According to the guided search theory^37^, visual search through pre-attentive processing can be accomplished in approximately 200–250 ms by looking at elements. In contrast to the slower, serial pattern of normal visual search, pre-attentive processing enables an almost instantaneous and parallel pattern of visual search.

Target detection tasks, which involve finding a target that differs in some low-level visual features among multiple distractors, are known to trigger pre-attentive processing^38^. When performing such tasks, our eyes can immediately detect the target when there is a difference in the low-level visual features between a distractor and a target, leading to the occurrence of pre-attentive processing. For example, when a target object differs from surrounding distractors in a specific low-level visual feature, such as being a unique shape or color, the eyes can quickly identify it through pre-attentive processing. These low-level visual features that trigger pre-attentive processing are known as pre-attentive visual features. Healey *et al*.^39^ have comprehensively categorized these features, demonstrating their utility in visual search tasks.

While pre-attentive visual features have been widely studied for their ability to enhance rapid visual information processing, their application in gaze-based authentication remains largely unexplored. Instead, prior research has predominantly focused on leveraging these features in areas such as data visualization and decision support. For example, Healey *et al*.^39^ employed pre-attentive visual features to visualize multivariate data elements, enabling simultaneous information presentation. Doerr *et al*.^40^ employed pre-attentive visual features to direct visual attention to specific areas of a Virtual Reality (VR) environment, and Kunze *et al*.^41^ applied them to visualize uncertainty in autonomous driving systems using Augmented Reality (AR). Additionally, some researchers have explored the impact of pre-attentive visual features on peripheral vision when displayed on large screens^42,43^.

These studies demonstrate the versatility of pre-attentive visual features in enhancing visual tasks across various domains. However, their potential to enable secure and efficient interaction paradigms, such as gaze-based authentication, has not been fully explored. To address this gap, we proposed the stimuli design and dataset for authentication based on the pre-attentive processing. By utilizing visual features that trigger pre-attentive responses, we aimed to facilitate swift visual searches within intricate static images. In order to intensify the complexity of the visual search task, we tasked participants with pinpointing all salient visual elements among numerous distractors.

## Methods

### Dataset design

In this study, visual stimuli were designed with pre-attentive processing that combines different visual elements to induce a user’s unique gaze pattern. To collect an involuntary response, visual stimulation for a short period of time within one second was presented. As mentioned in the background section, we designed these visual stimuli to be complex, goal-directed, static, and to induce pre-attentive processing. While the complexity of static visual stimuli has been shown to correlate with authentication accuracy^17^, limited research exists on the specific factors that define the complexity of stimuli. In the previous studies, the complexity of static stimuli is often characterized by the number of elements visible and the variety of low-level visual features that compose those elements^25,33^.

**Fig. 1:**
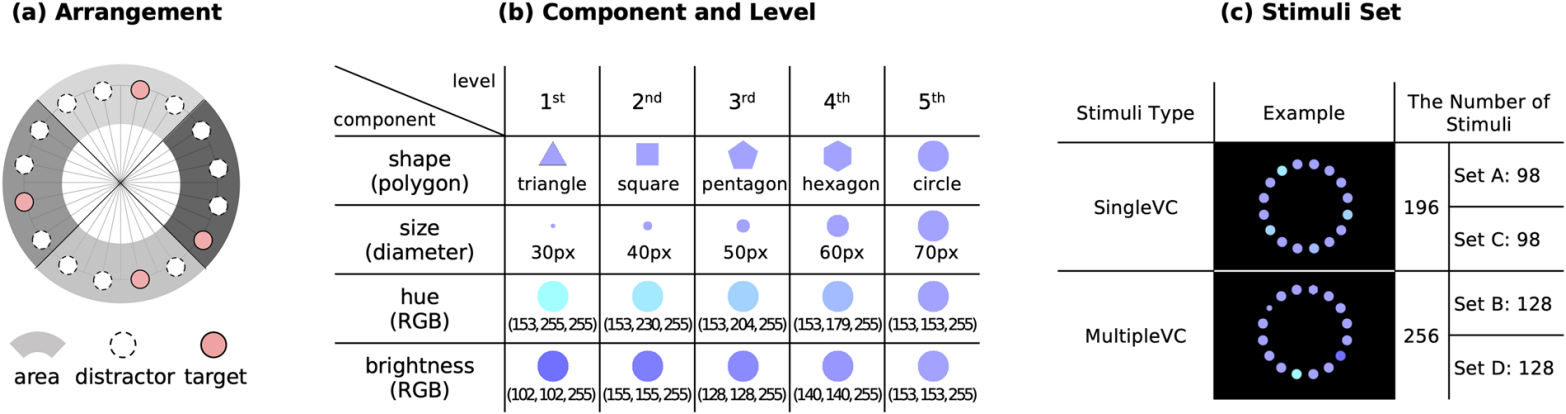
Criteria for design of visual stimuli; (**a**) Arrangement, (**b**) Component and Level, (**c**) Stimuli Set.

In this study, we aimed to design visual stimuli that were complex to enable visual search through pre-attentive processing. However, there is a trade-off between the complexity and effectiveness of pre-attentive processing. Pre-attentive processing—the ability to instantly distinguish target from surrounding distractors—requires less complexity for this phenomenon to be prominent. To balance these factors, we used a multiple target detection task as our stimuli. Figure 1 shows the criteria for the design of the visual stimuli. The stimuli were organized with four target elements positioned at the top, bottom, left, and right, and with various levels of pre-attentive visual features. The user was asked to visually detect these targets with anomalous characteristics compared to those of the distractors, which are the default elements making up the majority of visual stimuli.

### Arrangement

Our visual stimuli were constructed as shown in Fig. 1(a) with 16 elements evenly distributed in a circular array (spaced 22.5° apart). The circular arrangement was employed to eliminate the effect of the distance of each element from the center as a user observes the stimuli. Up to four targets in a stimulus were placed in all areas to prevent the skewness of the targets so that the user’s gaze was evenly distributed across the visual stimuli.

### Component and Level

Visual component (VC) consists of four types: shape, size, hue, and brightness, which are pre-attentive visual features commonly used in related studies^44–46^. As shown in Fig. 1(b), each VC has five intensity levels. Among these levels, the distractor was set to the fifth level, and the target was created by changing the level of the VC.

### Stimuli Set

Stimuli are divided into two types: SingleVC, with only one type of VC as the target, and MultipleVC, containing four types of VCs. The difference between MultipleVC and SingleVC is whether or not different VCs are used to construct the target. In SingleVC, the same VCs are used, but the targets are assigned to a random level different from the distractor, while in MultipleVC, the four types of the 16 elements presented in the visual stimuli, any elements that are not targeted acts as distractors. As previously noted, these distractors constitute the fifth-level component and exhibit a uniform appearance. Given the number of cases for each VC, the total number of SingleVCs was 196 (Sets A and C) and a total of 256 MultipleVCs (Sets B and D), with a level for each of the four VCs. The stimuli set was counterbalanced by combining different visual components, levels, locations, and numbers of targets, and randomizing the sequence to present these stimuli. This was aimed at minimizing the learning effect of users on the prediction of certain targets, with the aim of solely collecting the gaze responses to random stimuli. Examples of SingleVC and MultipleVC stimuli are shown in Fig. 1(c). In Appendix Figure A1 and Figure A2, we described the methodology for establishing the total number of each VC.

### Participants

The data were collected from 34 people, consisted of 20 males and 14 females. The subjects were recruited through the advertisement of the data collection on the college community website. Their average age was 22.7 with a standard deviation (std) of 4.20 (spanned from 17 to 33). The participants were allowed to wear glasses or contact lenses during the experiment under the instruction to maintain a comfortable state throughout data collection. Appendix Table A1 provides the demographic information of participants, including their vision correction methods and corrected visual acuity. After the completion of the experiment, they received 60 K KRW (equivalent to 45.89 USD) for their participation. This research was conducted with the approval of the Institutional Review Board (IRB) of Gwangju Institute of Science and Technology under the protocol code 20221201-HR-69-04-02 on December 21, 2022. All participants were adequately informed about the purpose, methods, potential risks, and benefits, and their voluntary consent was obtained in writing. All participants consented to data disclosure, and data for all subjects were included in our dataset.

### Data collection procedure

**Fig. 2:**
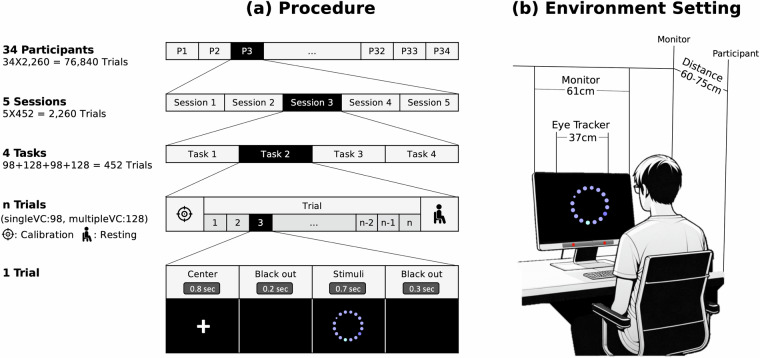
Experimental procedure of a task and environment setting.

The methodology for how to present the designed visual stimuli to the user to collect responses is shown in Fig. 2(a). Each trial to provide the designed stimuli consisted of the following sequence: center adjustment, blackout, stimuli, and blackout again. Each step was displayed for 0.8, 0.2, 0.7, and 0.3 s in a row, so it took approximately 2 s to collect gaze information for one stimulus. The experiment spanned for five days, with each participant engaging in a single session each day. Participants were instructed to visually detect the anomalous elements (targets) from given stimuli, and were guided with an illustrating example of the stimuli. The total number of samples to collect in a session was 452 stimuli (196 SingleVC + 256 MultipleVC). As showing 452 stimuli continuously would loose their attention and increase eye fatigue, we divided the task into four stimuli sets, consisting of half SingleVC and half MultipleVC stimuli. Stimuli sets A and C consist of 98 stimuli each, whereas stimuli sets B and D consist of 128 stimuli each (see Fig. 2(a)). During the task, the gaze calibration, trial, and rest phase were performed sequentially, as depicted in Fig. 2(a). There were four tasks per session, and in each task, one stimuli set was presented in a randomized order during the trial task phase. Before the initiation of each task set, a gaze calibration was performed on the participants, and they rested for 3 min between tasks. To briefly outline the procedure, SingleVC and MultipleVC simulation sets were alternated, and the order of task sets was counterbalanced for five sessions. A total of 76,840 gaze samples (34 participants x 5 sessions x (2 tasks x 98 SingleVC stimuli + 2 tasks x 128 MultipleVC stimuli)) were collected.

### Environment setting

The eye tracking results were extracted using the remote eye tracker (Tobii Pro Fusion), which detects eye movements in real time through an optical sensor using a high-resolution camera and infrared light^47^. The horizontal and vertical viewing angles of the traceable area were approximately 120° and 90°, respectively. The sampling frequency per second was up to 120 Hz. Information, such as gaze point (x and y coordinates) and pupil size, were extracted using Tobii Pro Lab software. The recording was made in a quiet laboratory room under uniform lighting conditions. The physical experimental environment for data collection is illustrated in Fig. 2(b). The monitor size and the stimuli size were adjusted in consideration of the field of view (13°)^48^, so that the pre-attentive processing can be performed at a glance. Based on these considerations, stimuli with a diameter of 21 cm were presented while maintaining a distance of 60–75 cm from a 27-inch sized FHD (Full High Definition) monitor, resulting in a field of view ranging from 7.47 to 9.92°.

## Data Records

**Fig. 3:**
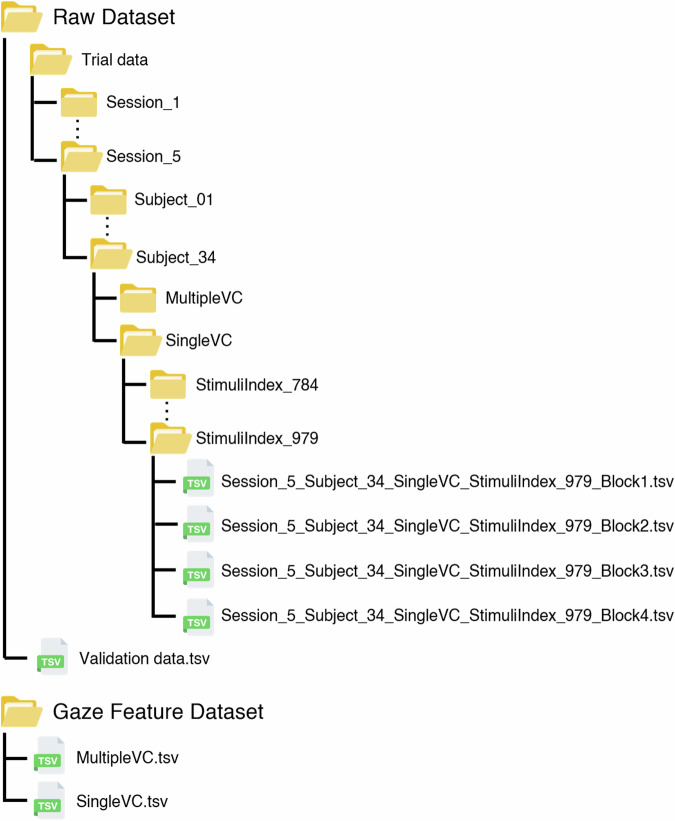
Organization of Raw and Gaze Feature Datasets.

Pre-AttentiveGaze dataset is available for download on figshare^49^. The dataset has a Creative Commons Attribution 4.0 International (CC BY 4.0) license. As illustrated in Fig. 3, the dataset is divided into two types: the raw dataset and the gaze feature dataset. The raw dataset contains trial data and validation data, while the gaze feature dataset is extracted from the trial data for the purpose of modeling the classification of participants. The gaze feature dataset includes two tsv files, “MultipleVC.tsv” and “SingleVC.tsv”, containing gaze feature data corresponding to their respective stimuli types.

### Raw dataset

Trial data is organized in a hierarchical directory structure, with the following categories: session, participant, stimuli type, and stimuli index. The stimuli type category serves to distinguish between MultipleVC and SingleVC data. The stimuli index identifies the specific stimuli employed, along with associated information such as arrangement, visual component, and level of the stimuli. The actual data in the directory is stored as four tsv files, each containing eye movement data from four blocks (center, blackout, stimuli, blackout) of the trial containing the stimuli. The names of the tsv files are converted to “Session_s_Subject_xx_t_StimuliIndex_i_b.tsv” as illustrated in the Table 1.

**Table 1:** Description of the trial data file naming convention.

Naming Parameter	Definition	Valid Values
s	session number	1–5
xx	participant ID	01–34
t	stimuli type	MultipleVC, SingleVC
i	stimuli index number	for MultipleVC: 0-1279, for SingleVC: 0–979
b	block	Block1(center), Block2(blackout), Block3(stimuli), Block4(blackout)

The columns in the tsv file for trial data are described in Table 2. This data records 19 details including x and y coordinates, position, pupil size, and fixation point. The contents is based on the Tobii Pro Lab User’s Manual (https://go.tobii.com/Tobii-Pro-Lab-data-export-info). In the dataset, missing values are represented by empty cells. These missing values were caused by factors such as blinking of eyes and temporary errors in the gaze tracker.

**Table 2:** Column description of the trial data file.

Category	Column	Unit	Description
Recording timestamp	Recording timestamp	*μ*s	Timestamp counted from the start of the recording
Gaze point 2D	Gaze point X Gaze point Y	pixel	Raw gaze coordination for both eyes combined
Pupil diameter	Pupil diameter left Pupil diameter right	mm	Estimated size of the eye pupil
Pupil diameter filtered	Pupil diameter filtered	mm	Pupil diameter after applying pupil diameter filter(noise reduction by moving median)
Validity of eye data	Validity left Validity right	Valid Invalid	Indication whether the eyes have been correctly identified
Eye position	Eye position left X Eye position left Y Eye position left Z Eye position right X Eye position right Y Eye position right Z	mm	3D position of the eyes
Eye movement type	Eye movement type	Fixation/Saccade/Unclassified/EyesNotFound	Type of eye movement event classified by the I-VT(velocity threshold identification) filter applied during the gaze data export
Gaze event duration	Gaze event duration	ms	Duration of the currently active eye movement
Eye movement type index	Eye movement type index	number	Order number in which an eye movement was recorded
Fixation point	Fixation point X Fixation point Y	pixel	Coordination of the fixation point.

The validation data is collected during the gaze calibration phase to evaluate the effectiveness of the calibration process and the reliability of participant’s eye movement data. The validation data includes two key parameters: average validation accuracy, and average validation precision. These parameters, as shown in Table 3, are measured in pixels. Average validation accuracy refers to the average value of the discrepancy between the actual gaze position and the position recorded by the eye tracker during the calibration phase^50^. Consequently, a lower average validation accuracy value indicates a smaller deviation in gaze prediction. Average validation precision represents the eye tracker’s reliability in reproducing consistent gaze point measurements across samples. It is calculated from the root mean square (RMS) of the sampled points during the gaze calibration phase. A lower average validation precision value signifies a smaller variation in gaze prediction.

**Table 3:** Column description of the validation data file.

Column	Description
Participant	ID for the participant who performed that calibration phase
Session	The session number during which the calibration phase was conducted
Stimuli type	The stimuli type presented after the calibration phase
List of stimuli index number	List of the stimuli indices presented after the calibration phase
Average validation accuracy	The average deviation between the predicted gaze points and the actual gaze targets
Average validation precision RMS	The RMS deviation of the predicted gaze points from their mean.

### Gaze feature dataset

In order to model identification from the collected data, the eye movement data collected in the stimuli block was processed with the following gaze features. Table 4 describes the gaze features investigated in this paper, commonly used in existing studies^17,20,22,28,30,51,52^. The gaze features were categorized into six types: Raw Gaze, Eye Movement, Fixation, Saccade, MFCC, Pupil. In addition to the gaze features in Table 4, the dataset includes participant, session, stimuli index information for the gaze samples used to extract these features.

**Table 4:** Gaze features used in the dataset.

Features	Description
**Raw Gaze**	
X	Corresponding to Gaze point X (*x*_1_, …, *x*_84_) in Table 2
Y	Corresponding to Gaze point Y (*y*_1_, …, *y*_84_) in Table 2
**Eye Movement**	
Path length	Length of path traveled in screen, computed as path length = ${\sum }_{i\mathrm{=1}}^{N-1}\sqrt{{({x}_{i+1}-{x}_{i})}^{2}+{({y}_{i+1}-{y}_{i})}^{2}}$
Gaze velocity	Velocity of path traveled in screen, computed as gaze velocity = $\frac{\sqrt{{(\varDelta x)}^{2}+{(\varDelta y)}^{2}}}{\varDelta t}$, $\varDelta t$ = 0.0083 sec
Gaze angle	Angular changes between consecutive raw gaze points
Eye movement type	Type of eye movement event classified by the I-VT filter (see Table 2); Fixation: 1, Saccade: 2, else: 0
**Fixation**	
Reaction time	Time until the first fixation is made outside the cross point (equal to the first fixation time)
Fixation duration	Duration per fixation interval
Fixation dispersion	Spatial spread during a fixation, computed as fixation dispersion = $(max(X)-min(X))+(max(Y)-min(Y))$
Fixation count	Number of fixation intervals identified within 84 sampled gaze points
**Saccade**	
Saccade duration	Duration per saccade interval
Saccade amplitude	The angular distance traveled by the eye during a saccadic movement, calculated as the angle between vectors from the eye’s position to fixation points before and after the saccade
Saccade velocity	The angular speed of the eye during a saccade, calculated by dividing the saccade amplitude by its duration
Saccade dispersion	Spatial spread during a saccade, computed as saccade dispersion = $(max(X)-min(X))+(max(Y)-min(Y))$
Saccade count	Number of saccade intervals identified within 84 sampled gaze points
**MFCC**	12 Mel-frequency cepstral coefficients for overall stimuli
**Pupil**	
Left pupil diameter	Pupil diameter of left eye
Right pupil diameter	Pupil diameter of right eye
Filtered pupil diameter	Pupil diameter after applying pupil diameter (see Table 2)

Before extracting gaze features from the raw dataset, we performed interpolation on unmeasured gaze data to facilitate accurate and consistent feature calculation. In the case of unmeasured gaze data, which are caused by factors such as blinking of eyes and temporary errors in the gaze tracker, linear interpolation, backward fill, and forward fill, which are commonly used in other studies^53,54^, were applied sequentially.

### Raw gaze

In our dataset, raw gaze corresponds to Gaze Point 2D of the trial data, consisting of x-axis data and y-axis data. It was collected from 0.7-second blocks of stimuli with an eye tracker at 120 Hz, with each block containing 84 samples (120 Hz × 0.7 s = 84 samples).

### Eye movement

Eye movement represents the motion of raw gaze data. Path length refers to the total distance traveled by the 84 gaze points, calculated by summing the distances between consecutive points. Gaze velocity is a list of eye movement velocities per frame, containing 83 samples, as the time interval ($\varDelta t$) between frames is 0.0083 seconds (1/120 of a second). Gaze angle represents the angular changes in direction between consecutive gaze points, normalized to capture shifts in movement, and is also represented with 83 samples. Eye movement types remain the same as described in Table 2, but the labeling has been adjusted: fixation is labeled as 1, saccade as 2, and other types as 0.

### Fixation

Fixation refers to the stabilization of gaze on a specific location for a period of time, providing critical information about visual attention and cognitive processes^55^. In our dataset, fixation-related features were extracted to characterize these periods of stability. Reaction time captures the latency until the first fixation outside the central cross point, serving as an indicator of participants’ response initiation. Fixation duration measures the time spent (milliseconds) on each fixation interval. Similarly, fixation dispersion, calculated for each fixation interval, quantifies the spatial spread of gaze points by measuring the range of movement in both the horizontal (x-axis) and vertical (y-axis) directions. Fixation dispersion determines the difference between the maximum and minimum gaze positions along each axis and sums these values to represent the overall spatial variability within the fixation interval. Both fixation duration and fixation dispersion share the same sample count, equal to the fixation count.

### Saccade

Saccades are rapid, ballistic eye movements that shift the gaze from one fixation point to another, providing insights into visual exploration and attention dynamics^56^. In our dataset, saccade-related features were extracted to characterize these transitions. Saccade duration measures the time taken (milliseconds) for each saccadic movement. Saccade amplitude quantifies the angular distance traveled by the eye during a saccade, calculated as the angle between vectors extending from the eye’s position to the fixation points before and after the saccade. Saccade velocity represents the angular speed of the eye during a saccade, derived by dividing the saccade amplitude by its duration. Saccade dispersion, calculated for each saccade, measures the spatial spread of gaze points during the movement by summing the range of movement along the horizontal (x-axis) and vertical (y-axis). Importantly, the sample counts for saccade duration, saccade amplitude, saccade velocity, and saccade dispersion are all equal to the saccade count, representing the total number of saccades identified in the dataset.

### MFCC

MFCCs (Mel-Frequency Cepstral Coefficients) are a compressive representation of spectral features derived from a signal and are traditionally used in applications such as speech recognition^57^. Nguyen *et al*.^20^ utilized MFCCs to encode features of eye-tracking data for gaze-based authentication. According to their study, the MFCC extraction process involves segmenting the signals into overlapping frames by applying a Hamming window, computing the short-term Fourier transform, mapping to the Mel scale, and applying a discrete cosine transform to produce a set of coefficients. These steps resulted in a noise-robust and concise representation of the temporal dynamics of the eye movement data, which improved recognition performance. In our study, we computed MFCCs in this way and used them to model eye movement characteristics by embedding them in gaze features.

### Pupil

Pupil size has been widely studied as an indicator of cognitive effort, arousal, and attentional states, making it a relevant physiological signal^58–61^. Although it is subject to confounding factors such as environmental light conditions and the relatively slow pupillary response time (occurring over hundreds of milliseconds), its integration into the dataset allows for exploratory analyses that combine gaze dynamics with physiological measurements.

Pupil size data, including left and right pupil diameters as well as filtered pupil diameters, has been incorporated into this dataset as an additional feature to complement eye movement metrics. While pupil size is not fully aligned with pre-attentive gaze research due to its slower temporal dynamics, its inclusion was motivated by its potential to enhance recognition performance in conjunction with eye movement features. Preliminary evaluations conducted in this study suggest that the addition of pupil size data can improve recognition performance, particularly when paired with other gaze-related features. This observed benefit justifies its inclusion, even though the primary focus of the research remains on eye movement.

## Technical Validation

### Overview of gaze data according to visual stimuli

**Fig. 4:**
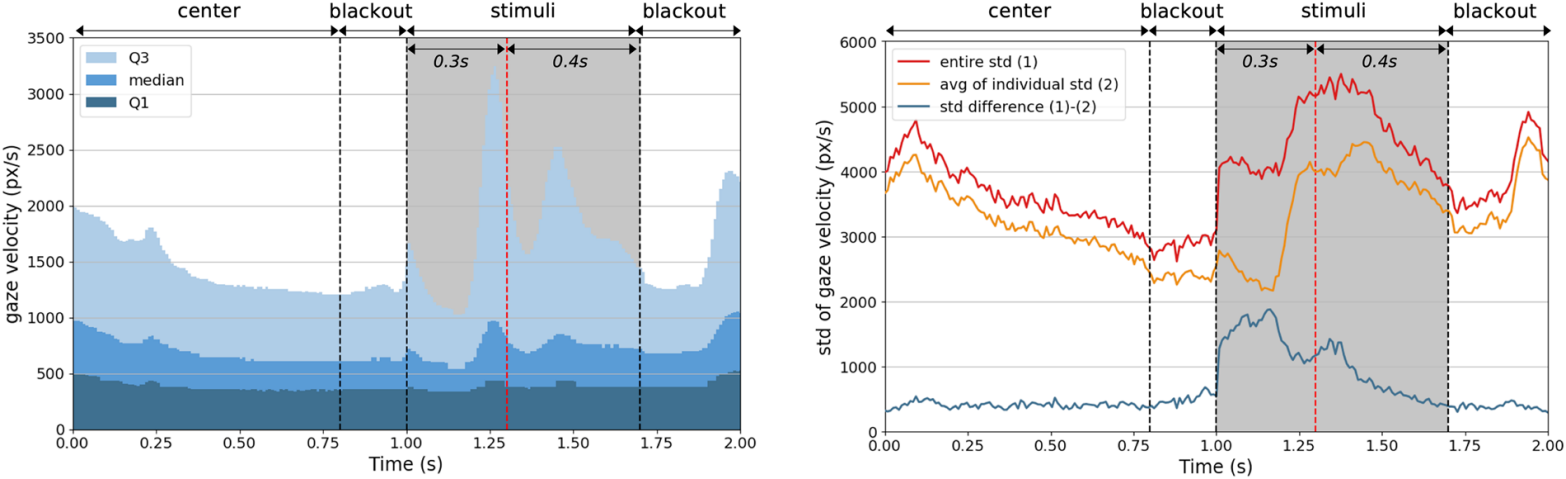
Trends in the overall gaze velocity during the trials (center adjustment, blackout, stimuli, blackout) of entire stimuli; left: interquartile range of gaze velocity, right: standard deviations (stds) of gaze velocity.

We first explored the patterns of visual interactions during the presentation of visual stimuli. Figure 4 illustrates the trends of the participants’ gaze velocity, calculated as described in Table 4, during trials (comprising center adjustment, blackout, stimuli, and another blackout as presented in Fig. 2(a)) with the entire stimuli set (MultipleVC and SingleVC). The graphs are segmented into four portions by black dotted lines, each representing a distinct phase of the trial, while the period exposing stimuli is highlighted as gray background. Figure 4(left) shows the first quartile (Q1), median, and third quartile (Q3) values of gaze velocities across all participants during the trial. As shown in the Fig. 4(left), Gaze velocity dynamically changes during the stimuli phase compared to other phases. This points to a heightened frequency of eye movements when viewing the stimuli, hinting at intricate gaze interactions induced by our stimuli compared to other segments of the trial. In fact, saccades occurred an average of 3.50 times (std 2.00) for SingleVC and 3.50 times (std 1.97) for MultipleVC during the 0.7 second stimulus interval. It is noteworthy that a rapid fluctuation in gaze velocity occurred within 0.3 seconds of stimulus onset (red dashed line in the graph). This indicates that our stimuli elicited pre-attentive processing.

Figure 4 (right) displays the std of gaze velocities, presenting three key data points: the std of gaze velocities for the entire participant pool (entire std), the average of the std values calculated from gaze velocities for each participant (avg of individual std), and the difference between these two stds (std difference). Remarkably, entire std was significantly heightened during the stimuli phase, even exceeding the values observed in the third quartile (see Fig. 4 (left)). This pattern indicates that our stimulus generated a divergent range of gaze velocities compared to other phases. Moreover, avg of individual std consistently recorded lower values than entire std, implying that gaze velocities exhibited by individual participants are more homogeneous compared to the overall population. This degree of homogeneity is most pronounced during the stimuli phase. Collectively, these findings imply that our stimuli effectively induced a wide spectrum of gaze velocities across individuals, facilitating the accumulation of a rich dataset capable of distinguishing between individual participants.

**Fig. 5:**
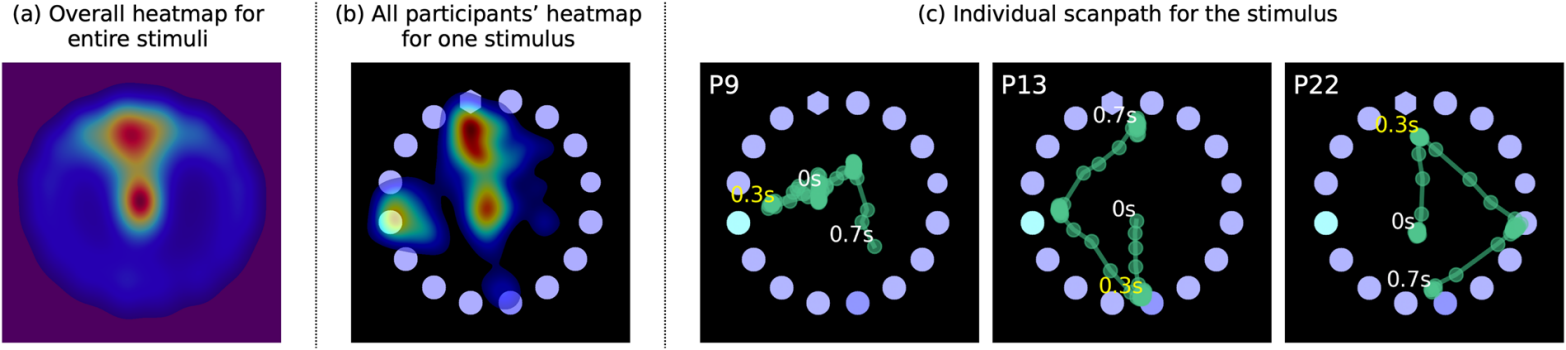
(**a**) Heatmap of raw gaze points across entire participants with overall stimuli, (**b**) Aggregated participants’ heatmap for one MultipleVC stimulus, (**c**) scanpath for the specific participants (P9, P13, and P22) of the MultipleVC stimulus.

The heatmap in Fig. 5(a) illustrates the distribution of raw gaze points across the 34 participants while they engaged with the overall stimuli including MultipleVC and SingleVC. As a consequence of the center adjustment implemented before the initiation of the stimuli, a concentration of raw gaze points in the center of the stimuli was noted. Subsequently, a general tendency emerged where participants often focused their gaze towards the top of the stimuli. Apart from the upper portion, the gaze was evenly distributed across other areas of the stimuli. The heatmap and scanpath samples reveal the raw gaze data of participants interacting with a single MultipleVC stimulus. As Shown in the heatmap of Fig. 5(b), it is apparent that the participants’ gaze predominantly focused on the area where the target element was located. As illustrated by individual scanpaths in Fig. 5(c), a significant majority initiated their first gaze movements in proximity to the target element within a span of 0.3 seconds. Thereafter, as participants attempted to locate other target elements, numerous gaze shifts were observed. The rapid initial movements towards the vicinity of the target element within the 0.3-second window highlight the influence of pre-attentive processing. This behavior was similarly observed for SingleVC stimuli.

### Preliminary learning pipelines

**Fig. 6:**
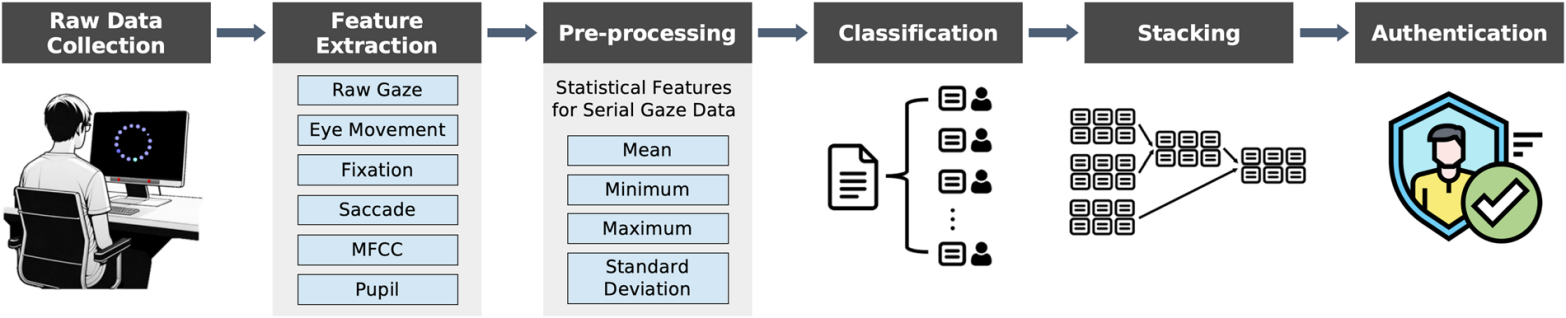
System architecture pipeline.

We propose an initial pipeline for exploitation, with the objective of facilitating more effective utilization of our data, as shown in Fig. 6. This pipeline involves raw data collection, gaze feature extraction, preprocessing for data refinement, and gaze-based authentication through classification using both single-sample and multiple-sample approaches. Evaluating the performance of gaze-based authentication through classification is a commonly used approach in previous studies^20,62–64^. Performance is assessed by measuring classification accuracy, which is calculated by dividing the gaze feature dataset into training and testing subsets and applying basic machine learning models such as Support Vector Machines. Given that the Pre-AttentiveGaze dataset was collected across multiple sessions, leave-one-session-out cross-validation was employed to account for potential temporal effects. Furthermore, we propose a method for aggregating our data samples and reporting the improvement in authentication performance.

### Preprocessing

Six gaze features (fixation duration, fixation dispersion, saccade duration, saccade velocity, saccade amplitude and saccade dispersion) have variable sample counts across gaze sequences due to differing fixation and saccade conditions in each sequence. To transform these variable-length features into a fixed-length feature vector suitable for a basic machine learning model, we computed a set of statistical features (mean, minimum, maximum, and standard deviation) for each feature, following the approach of Pfeuffer *et al*.^65^. After obtaining the fixed-length representation, we applied z-score normalization to ensure that all gaze features are on the comparable scale. Specifically, for each gaze feature the following transformation was used:

$$z=\frac{x-\mu }{\sigma },$$

where μ and σ represent the mean and standard deviation calculated from the train data. In our leave-one-session-out cross-validation scheme, the test data was normalized using the corresponding train data’s statistics (*μ* and *σ*), thereby preventing any leakage of information from the test data into the training process. Samples containing the missing data were excluded from the dataset in order to model it.

### Classification results

The classification results were derived using the leave-one-session-out cross-validation method as shown in Table 5. The train data was constructed from four sessions and the test data was comprised of the remaining session. As our dataset is consisted of 5 sessions, the cross-validation is conducted in a five-fold manner. All data were preprocessed as detailed in the Preprocessing section.

**Table 5:** Mean (standard deviation) of classification results by leave-one-session-out cross-validation on all gaze features.

Method	MultipleVC				SingleVC			
	Accuracy	Precision	Recall	F1	Accuracy	Precision	Recall	F1
ZeroR	0.034 (0.001)	0.001 (0.000)	0.034 (0.001)	0.002 (0.000)	0.033 (0.000)	0.001 (0.000)	0.033 (0.000)	0.002 (0.000)
DT	0.423 (0.067)	0.429 (0.065)	0.423 (0.067)	0.419 (0.066)	0.418 (0.040)	0.424 (0.038)	0.418 (0.040)	0.414 (0.039)
KNN	0.453 (0.073)	0.486 (0.072)	0.453 (0.073)	0.442 (0.072)	0.433 (0.043)	0.465 (0.046)	0.433 (0.043)	0.421 (0.044)
NB	0.396 (0.055)	0.418 (0.061)	0.396 (0.055)	0.380 (0.052)	0.400 (0.035)	0.420 (0.041)	0.400 (0.035)	0.380 (0.034)
SVM	0.682 (0.082)	0.689 (0.082)	0.682 (0.082)	0.677 (0.082)	0.667 (0.058)	0.674 (0.060)	0.667 (0.058)	0.661 (0.060)
LR	0.661 (0.067)	0.664 (0.068)	0.661 (0.067)	0.656 (0.068)	0.659 (0.051)	0.662 (0.054)	0.659 (0.051)	0.653 (0.053)
RF	0.613 (0.085)	0.619 (0.087)	0.613 (0.085)	0.603 (0.086)	0.605 (0.053)	0.604 (0.056)	0.605 (0.053)	0.591 (0.056)

Six classifiers provided by the scikit-learn library, a widely used open-source machine learning framework, were employed in this study. The classifiers included decision tree (DT), k-nearest neighbor (kNN), naïve Bayes (NB), support vector machine (SVM), logistic regression (LR), and random forest (RF). Each classifier was configured with parameters suitable for general-purpose classification tasks.

For the DT classifier, no limit was imposed on the depth of the tree, and the minimum number of samples required to split an internal node was set to two. The kNN classifier used five neighbors with uniform weights for predictions. The NB classifier followed the Gaussian distribution, with default priors and a variance smoothing factor of 10^−9^. The SVM classifier was configured with a radial basis function (RBF) kernel, a regularization parameter *C* of 1.0, and enabled probability estimates. The LR classifier used *L*_2_ regularization with a regularization strength *C* of 1.0. Lastly, the RF classifier consisted of 100 estimators, used the Gini impurity criterion for node splitting, and did not impose a maximum depth for the trees. Any unspecified parameters for these classifiers were left at their default values as defined in the scikit-learn library.

In the classification of 34 individuals based on a single sample including 84 gaze points within a 0.7-second block of stimuli, the SVM achieved the highest accuracy of 0.682 and 0.667 on the MultipleVC and SingleVC datasets, respectively. These values represent a 64.8%p and 63.4%p improvement over the baseline, ZeroR, respectively. Meanwhile, as shown in Appendix Table A2, the classification performance was evaluated excluding pupil-related gaze features. The highest accuracy for MultipleVC was achieved by SVM at 0.516, while the highest accuracy for SingleVC was achieved by RF at 0.489. When pupil-related gaze features were included, there was a notable performance improvement of 16.4%p for MultipleVC and 17.8%p for SingleVC, confirming their meaningful contribution to enhancing overall performance.

### Classification results by sample aggregation

We assumed a scenario in which the prediction is conducted through the processing of multiple samples. In this scenario, the outputs of the SVM classifier, chosen for its high performance in single-sample classification, from each of the multiple samples are aggregated to derive the final prediction score. We performed multiple sample aggregation according to the following algorithm.

Each sample *x*_*i*_ is passed through the classifier to obtain a probability distribution over *C* classes (users). Let *f*(*x*_*i*_) represent the classifier function. Then the output for each sample *i* is:

1 $${p}_{i}=f({x}_{i})=[{p}_{i\mathrm{,1}},{p}_{i\mathrm{,2}},\ldots ,{p}_{i,C}]$$

Here, *p*_*i,j*_ is the predicted probability that sample *i* belongs to class *j*. For stack-*N* aggregation, we combine the prediction probabilities of all N samples by elementwise multiplication of their probability vectors. The aggregated prediction vector *p*_agg_ is computed as follows:

2 $${p}_{{\rm{agg}}}=\underset{i=1}{\overset{N}{\odot }}{p}_{i}=[\mathop{\prod }\limits_{i=1}^{N}{p}_{i,1},\mathop{\prod }\limits_{i=1}^{N}{p}_{i,2},\ldots ,\mathop{\prod }\limits_{i=1}^{N}{p}_{i,C}]$$

The final prediction label is obtained by applying the argmax function to the aggregated prediction vector *p*_agg_:

3 $${\rm{prediction}}\,{\rm{output}}={\rm{argmax}}({p}_{{\rm{agg}}})$$

**Fig. 7:**
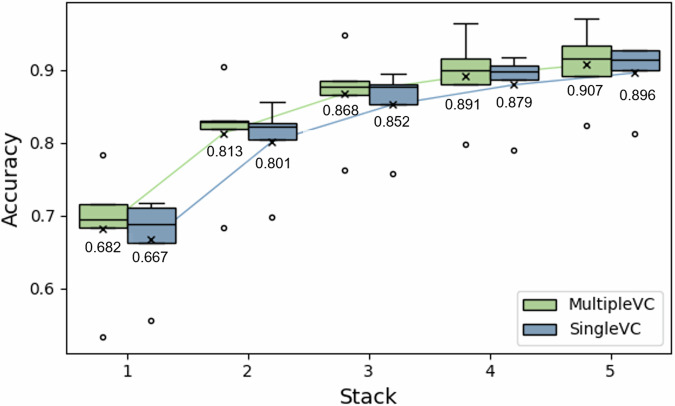
Classification accuracies from SVM with a change in the number of stacked samples in prediction.

In case of aggregating 3 samples (stack-3), this is technically the result of analyzing 2.1 seconds of gaze data, which is 0.7 seconds of three gaze data pooled together. Figure 7 shows the improvement in prediction accuracy with an increase in the number of samples used for prediction, where each boxplot represents the distribution of accuracies obtained through leave-one-session-out cross validation as same way as single sample classification. For MultipleVC, the accuracies for stack-1 to stack-5 were 68.2, 81.3, 86.8, 89.1, and 90.7%, respectively, and those for SingleVC were 66.7, 80.1, 85.2, 87.9, and 89.6%. Compared to that of the single-sample (stack-1) classification, a performance improvement of approximately 20%p was achieved when stack-3 was employed (with a total time of 0.7 s × 3 = 2.1 s). However, the increase in the accuracy for stack-4 (2.8 s) and stack-5 (3.5 s) was marginal, and appeared to converge.

The pipeline proposed in this paper includes a preliminary implementation that preprocesses the data to train a simple machine learning model and applies multiple sample aggregation. It is anticipated that further research will utilize this data in a more seamlessly integrated manner.

### Data loss

**Fig. 8:**
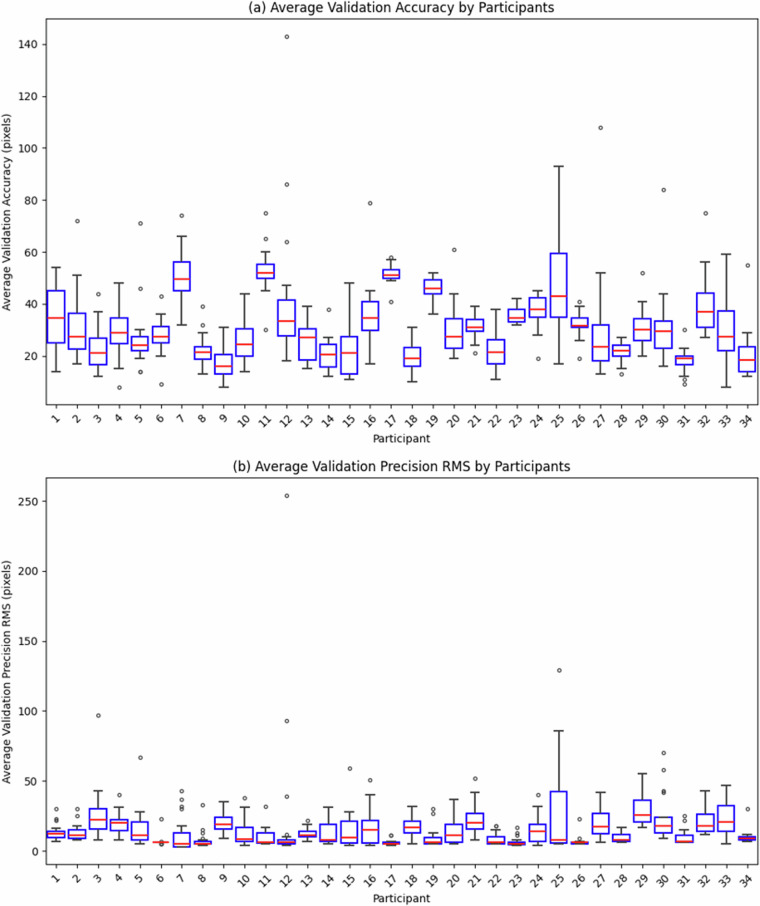
Distribution of validation data; (**a**) Average Validation Accuracy, (**b**) Average Validation Precision RMS.

The Pre-AttentiveGaze dataset contains the validation data as detailed in Table 3. The validation data represents the data loss that occurred during the eye tracking process, despite our efforts to maintain high data quality during the collection process. Figure 8 presents the average validation accuracy and average validation precision RMS for each participant in the validation data. The average validation accuracy had a mean value of 31.22 with a standard deviation of 14.33, while the average validation precision RMS had a mean value of 11.21 with a standard deviation of 16.82. In addition, there is no significant correlation between participants’ vision correction methods and their average validation accuracy or average validation precision RMS.

## Limitations and future work

### Investigating the effectiveness of visual components

Stimuli design affects how an individual’s gaze features are triggered^66^. The stimuli in this study were designed to induce pre-attentive processing while incorporating complex eye movement patterns. To achieve this, various VC conditions and levels were included. However, the effects of these VC conditions and levels on individual gaze features remain unexplored. Future research should examine how different stimuli, based on VC conditions, can effectively elicit gaze features that distinguish between individuals.

### Enhancing the versatility of gaze data collection

Our gaze data were collected in a controlled laboratory setting, an environment likely to produce relatively noise-free data. The experiments were conducted in a quiet room with consistent lighting conditions, and each task included a gaze calibration process lasting 1-2 minutes to ensure accurate data collection. As a result, it remains uncertain whether our visual stimuli would function effectively in less controlled or “in-the-wild” settings. Lighting conditions are known to affect pupil diameter^67^, and uncontrolled environments, such as outdoor settings or noisy locations, may introduce various events that influence cognitive processes and add noise to gaze behavior^68,69^. To address these limitations, future work should focus on developing sophisticated mechanisms to minimize the influence of equipment specifications and sampling data, or to verify the robustness of the model through data collected in different collection environments. Additionally, methods for gaze feature extraction that can mitigate the effects of noise should also be explored.

Another limitation lies in the demographic characteristics of the participants. The study’s participant pool was limited in both size and diversity. To evaluate the generalizability of our method, future studies should involve a larger number of participants, as well as individuals from a wider age range and diverse backgrounds.

### Designing advanced model architecture

Through the preliminary learning pipelines applied to this dataset, we confirmed its potential for modeling applications. However, our findings are based on basic machine learning models, and it is likely that more advanced architectures could significantly enhance performance. For instance, future research could investigate different model architectures for deeper modeling, such as temporal sequence modeling (e.g., LSTMs^70^) or spatial modeling with image-based inputs, such as gaze heatmap that capture attention to spatial regions (e.g., CNNs^71^).

### Concluding remarks

We propose the Pre-AttentiveGaze dataset, designed to enable gaze-based authentication using momentary eye movement information. This dataset was constructed by collecting 76,840 gaze samples from 34 participants over 5 sessions and includes both the raw dataset and extracted gaze feature dataset. Our stimuli design, which integrates insights from previous gaze-based authentication researches and the concept of pre-attentive processing, facilitated rapid eye movements. An analysis of gaze velocity confirmed that the stimuli effectively induced rich eye movements within the 0.7 s presentation window. Furthermore, the preliminary learning pipeline demonstrated the potential for utilizing the Pre-AttentiveGaze dataset and its associated stimuli design in future gaze-based authentication research, including scenarios that require analyzing momentary eye movements.

## Supplementary information

Supplementary Information

## Data Availability

The code utilized to extract the graphs and results presented in this paper is accessible on GitHub and may be accessed via the following link: (https://github.com/dynamic98/Pre-AttentiveGaze). In the case of the dataset, it was not possible to include it on GitHub due to limited capacity. Therefore, it can be downloaded from Figshare and transferred to the data folder in the GitHub directory. The code comprises a number of components, including code for the extraction of gaze features from raw data, code for the generation of the stimuli images used from the stimuli index, and code for the application of data to z-score normalization and machine learning. Additionally, the code includes the functionality to generate a heatmap and scanpath, as illustrated in Fig. 5. Researchers utilizing this code have the option of obtaining heatmaps and scanpaths for additional samples and participants.

## Appendix

**Stimuli design principle with formulas**

Based on the designed stimuli, number of visual component (*n*_*VC*_) is four, and number of level (*n*_*l*_) except for background is also four. Variables that change in some cases are the number of targets in stimuli (*t*) and degree (*d*), which is the number of levels of each target.

**Single visual component stimuli**

SingleVC stimuli consist of a single visual component with 16 elements and *t* ranges from 1 to 4. There are two cases of configure stimuli: levels of each target singular as Figure A1(i) and duplicated as Figure A1(ii).

As two cases, Figure A1(i) and (ii), are mutually exclusive events, the addition of two cases together completes formula (4).

4 $$SingleVC={n}_{VC}\times {\mathop{\sum }\limits_{t}^{4}}_{{n}_{l}}{{\rm{C}}}_{t}+{n}_{VC}\times {\mathop{\sum }\limits_{d}^{3}}_{4}{{\rm{C}}}_{d}{\times }_{d}{{\rm{H}}}_{\mathrm{(4}-d)}$$

**Multiple visual component stimuli**

MultipleVC stimuli consist of multiple visual component types. Each of the four types of visual component is selected as a target, so the number of targets *t* is also four. Each distractor can have a level of 1 to 4. Figure A2 shows examples of the MultipleVC stimuli. Considering the number of cases where one of the four levels is selected by the number of visual components, the following formula (5) is derived.

5 $$MultipleVC{=(}_{{n}_{l}}{{\rm{C}}}_{1}{)}^{{n}_{VC}}$$

**Demographic information about participants**

### Classification results excluding gaze features related to the pupil

This classification results were also derived using the leave-one-session-out cross-validation method. Pupil-related gaze features, such as Left pupil diameter, Right pupil diameter, and Filtered pupil diameter, are excluded during train and test dataset organization process. With the exception of the pupil-related gaze feature process, all processes were performed in the same manner as in Table 5.

## References

[1] Bradley, M. M., Miccoli, L., Escrig, M. A. & Lang, P. J. The pupil as a measure of emotional arousal and autonomic activation. *Psychophysiology* **45**, 602–607, 10.1111/j.1469-8986.2008.00654.x (2008). 18282202 10.1111/j.1469-8986.2008.00654.x PMC3612940

[2] Chen, S., Epps, J., Ruiz, N. & Chen, F. Eye activity as a measure of human mental effort in hci. In *Proceedings of the 16th International Conference on Intelligent User Interfaces*, IUI ‘11, 315–318, 10.1145/1943403.1943454 (Association for Computing Machinery, New York, NY, USA, 2011).

[3] Erbilek, M., Fairhurst, M. & Abreu, M. C. D. C. Age prediction from iris biometrics. In *5th International Conference on Imaging for Crime Detection and Prevention (ICDP 2013)*, 1–5, 10.1049/ic.2013.0258 (2013).

[4] Zhang, A. T. & Le Meur, B. O. How old do you look? inferring your age from your gaze. In *2018 25th IEEE International Conference on Image Processing (ICIP)*, 2660–2664, 10.1109/ICIP.2018.8451219 (2018).

[5] Abdi Sargezeh, B., Tavakoli, N. & Daliri, M. R. Gender-based eye movement differences in passive indoor picture viewing: An eye-tracking study. *Physiology & Behavior* **206**, 43–50, 10.1016/j.physbeh.2019.03.023 (2019). 30922820 10.1016/j.physbeh.2019.03.023

[6] Cantoni, V., Porta, M., Galdi, C., Nappi, M. & Wechsler, H. Gender and age categorization using gaze analysis. In *2014 Tenth International Conference on Signal-Image Technology and Internet-Based Systems*, 574–579, 10.1109/SITIS.2014.40 (2014).

[7] Chauhan, H., Prasad, A. & Shukla, J. Engagement analysis of adhd students using visual cues from eye tracker. In *Companion Publication of the 2020 International Conference on Multimodal Interaction*, ICMI ‘20 Companion, 27–31, 10.1145/3395035.3425256 (Association for Computing Machinery, New York, NY, USA, 2021).

[8] Liu, W. *et al*. Efficient autism spectrum disorder prediction with eye movement: A machine learning framework. In *2015 International Conference on Affective Computing and Intelligent Interaction (ACII)*, 649–655, 10.1109/ACII.2015.7344638 (2015).

[9] Prabha, A. J. & Bhargavi, R. Prediction of dyslexia from eye movements using machine learning. *IETE Journal of Research* **68**, 814–823, 10.1080/03772063.2019.1622461 (2022).

[10] Sun, J., Liu, Y., Wu, H., Jing, P. & Ji, Y. A novel deep learning approach for diagnosing alzheimer’s disease based on eye-tracking data. *Frontiers in Human Neuroscience* **16**, 10.3389/fnhum.2022.972773 (2022). 10.3389/fnhum.2022.972773 PMC9500464 36158627

[11] Jiang, H. *et al*. Dcamil: Eye-tracking guided dual-cross-attention multi-instance learning for refining fundus disease detection. *Expert Systems with Applications* **243**, 122889, 10.1016/j.eswa.2023.122889 (2024).

[12] Yang, H. *et al*. An automatic detection method for schizophrenia based on abnormal eye movements in reading tasks. *Expert Systems with Applications* **238**, 121850, 10.1016/j.eswa.2023.121850 (2024).

[13] Khushaba, R. N. *et al*. Consumer neuroscience: Assessing the brain response to marketing stimuli using electroencephalogram (eeg) and eye tracking. *Expert Systems with Applications* **40**, 3803–3812, 10.1016/j.eswa.2012.12.095 (2013).

[14] Peterson, J. *et al*. Understanding student success in chemistry using gaze tracking and pupillometry. In *Artificial Intelligence in Education*: *17th International Conference, AIED 2015, Madrid, Spain, June 22-26, 2015. Proceedings 17*, 358–366 (Springer, 2015).

[15] Hoppe, S., Loetscher, T., Morey, S. A. & Bulling, A. Eye movements during everyday behavior predict personality traits. *Frontiers in Human Neuroscience* **12**, 10.3389/fnhum.2018.00105 (2018). 10.3389/fnhum.2018.00105 PMC5912102 29713270

[16] Hoppe, S., Loetscher, T., Morey, S. & Bulling, A. Recognition of curiosity using eye movement analysis. In *Adjunct Proceedings of the 2015 ACM International Joint Conference on Pervasive and Ubiquitous Computing and Proceedings of the 2015 ACM International Symposium on Wearable Computers*, UbiComp/ISWC'15 Adjunct, 185–188, 10.1145/2800835.2800910 (Association for Computing Machinery, New York, NY, USA, 2015).

[17] Katsini, C., Abdrabou, Y., Raptis, G. E., Khamis, M. & Alt, F. The role of eye gaze in security and privacy applications: Survey and future hci research directions. In *Proceedings of the 2020 CHI Conference on Human Factors in Computing Systems*, CHI ‘20, 1–21, 10.1145/3313831.3376840 (Association for Computing Machinery, New York, NY, USA, 2020).

[18] Holland, C. & Komogortsev, O. V. Biometric identification via eye movement scanpaths in reading. In *2011 International Joint Conference on Biometrics (IJCB)*, 1–8, 10.1109/IJCB.2011.6117536 (2011).

[19] Bednarik, R., Kinnunen, T., Mihaila, A. & Fränti, P. Eye-movements as a biometric. In Kalviainen, H., Parkkinen, J. & Kaarna, A. (eds.) *Image Analysis*, 780–789 (Springer Berlin Heidelberg, Berlin, Heidelberg, 2005).

[20] Cuong, N. V., Dinh, V. & Ho, L. S. T. Mel-frequency cepstral coefficients for eye movement identification. In *2012 IEEE 24th International Conference on Tools with Artificial Intelligence*, vol. 1, 253–260, 10.1109/ICTAI.2012.42 (2012).

[21] Kasprowski, P. & Harezlak, K. Biometric identification using gaze and mouse dynamics during game playing. In Kozielski, S., Mrozek, D., Kasprowski, P., Małysiak-Mrozek, B. & Kostrzewa, D. (eds.) *Beyond Databases, Architectures and Structures. Facing the Challenges of Data Proliferation and Growing Variety*, 494–504 (Springer International Publishing, Cham, 2018).

[22] Kasprowski, P. & Ober, J. Eye movements in biometrics. In Maltoni, D. & Jain, A. K. (eds.) *Biometric Authentication*, 248–258 (Springer Berlin Heidelberg, Berlin, Heidelberg, 2004).

[23] Kinnunen, T., Sedlak, F. & Bednarik, R. Towards task-independent person authentication using eye movement signals. In *Proceedings of the 2010 Symposium on Eye-Tracking Research & Applications*, ETRA ‘10, 187–190, 10.1145/1743666.1743712 (Association for Computing Machinery, New York, NY, USA, 2010).

[24] Sluganovic, I., Roeschlin, M., Rasmussen, K. B. & Martinovic, I. Using reflexive eye movements for fast challenge-response authentication. In *Proceedings of the 2016 ACM SIGSAC Conference on Computer and Communications Security*, CCS ‘16, 1056–1067, 10.1145/2976749.2978311 (Association for Computing Machinery, New York, NY, USA, 2016).

[25] Song, C., Wang, A., Ren, K. & Xu, W. Eyeveri: A secure and usable approach for smartphone user authentication. In *IEEE INFOCOM 2016 - The 35th Annual IEEE International Conference on Computer Communications*, 1–9, 10.1109/INFOCOM.2016.7524367 (2016).

[26] Eberz, S., Rasmussen, K. B., Lenders, V. & Martinovic, I. Looks like eve: Exposing insider threats using eye movement biometrics. *ACM Trans. Priv. Secur*. **19**, 10.1145/2904018 (2016).

[27] Zhang, Y., Hu, W., Xu, W., Chou, C. T. & Hu, J. Continuous authentication using eye movement response of implicit visual stimuli. *Proc. ACM Interact. Mob. Wearable Ubiquitous Technol*. **1**, 10.1145/3161410 (2018).

[28] George, A. & Routray, A. A score level fusion method for eye movement biometrics. *Pattern Recognition Letters* **82**, 207–215, 10.1016/j.patrec.2015.11.020 (2016). An insight on eye biometrics.

[29] Griffith, H., Lohr, D., Abdulin, E. & Komogortsev, O. Gazebase, a large-scale, multi-stimulus, longitudinal eye movement dataset. *Scientific Data* **8**, 184 (2021). 34272404 10.1038/s41597-021-00959-y PMC8285447

[30] Lyamin, A. V. & Cherepovskaya, E. N. Biometric student identification using low-frequency eye tracker. In *2015 9th International Conference on Application of Information and Communication Technologies (AICT)*, 191–195, 10.1109/ICAICT.2015.7338544 (2015).

[31] Rigas, I., Economou, G. & Fotopoulos, S. Biometric identification based on the eye movements and graph matching techniques. *Pattern Recognition Letters* **33**, 786–792, 10.1016/j.patrec.2012.01.003 (2012).

[32] Seha, S., Papangelakis, G., Hatzinakos, D., Zandi, A. S. & Comeau, F. J. Improving eye movement biometrics using remote registration of eye blinking patterns. In *ICASSP 2019 - 2019 IEEE International Conference on Acoustics, Speech and Signal Processing (ICASSP)*, 2562–2566, 10.1109/ICASSP.2019.8683757 (2019).

[33] Yoon, H.-J., Carmichael, T. R. & Tourassi, G. Gaze as a biometric. In Mello-Thoms, C. R. & Kupinski, M. A. (eds.) *Medical Imaging**2014**:**Image Perception, Observer Performance, and Technology Assessment*, vol. 9037, 903707, 10.1117/12.2044303. International Society for Optics and Photonics (SPIE, 2014).

[34] Henderson, J. M. & Hollingworth, A. Eye movements and visual memory: Detecting changes to saccade targets in scenes. *Perception & psychophysics* **65**, 58–71 (2003). 12699309 10.3758/bf03194783

[35] Healey, C. & Enns, J. Attention and visual memory in visualization and computer graphics. *IEEE Transactions on Visualization and Computer Graphics* **18**, 1170–1188, 10.1109/TVCG.2011.127 (2012). 21788672 10.1109/TVCG.2011.127

[36] Celikors, E. & Wells, N. M. Are low-level visual features of scenes associated with perceived restorative qualities? *Journal of Environmental Psychology* **81**, 101800, 10.1016/j.jenvp.2022.101800 (2022).

[37] Wolfe, J. M. Guided search 2.0 a revised model of visual search. *Psychonomic bulletin & review* **1**, 202–238 (1994). 24203471 10.3758/BF03200774

[38] Healey, C. G., Booth, K. S. & Enns, J. T. High-speed visual estimation using preattentive processing. *ACM Trans. Comput.-Hum. Interact.* **3**, 107–135, 10.1145/230562.230563 (1996).

[39] Healey, C. & Enns, J. Large datasets at a glance: combining textures and colors in scientific visualization. *IEEE Transactions on Visualization and Computer Graphics* **5**, 145–167, 10.1109/2945.773807 (1999).

[40] Doerr, N., Angerbauer, K., Reinelt, M. & Sedlmair, M. Bees, birds and butterflies: Investigating the influence of distractors on visual attention guidance techniques. In *Extended Abstracts of the 2023 CHI Conference on Human Factors in Computing Systems*, CHI EA ‘23, 10.1145/3544549.3585816 (Association for Computing Machinery, New York, NY, USA, 2023).

[41] Kunze, A., Summerskill, S. J., Marshall, R. & Filtness, A. J. Augmented reality displays for communicating uncertainty information in automated driving. In *Proceedings of the 10th International Conference on Automotive User Interfaces and Interactive Vehicular Applications*, AutomotiveUI ‘18, 164–175, 10.1145/3239060.3239074 (Association for Computing Machinery, New York, NY, USA, 2018).

[42] Gutwin, C., Cockburn, A. & Coveney, A. Peripheral popout: The influence of visual angle and stimulus intensity on popout effects. In *Proceedings of the 2017 CHI Conference on Human Factors in Computing Systems*, CHI ‘17, 208–219, 10.1145/3025453.3025984 (Association for Computing Machinery, New York, NY, USA, 2017).

[43] Mairena, A., Gutwin, C. & Cockburn, A. Peripheral notifications in large displays: Effects of feature combination and task interference. In *Proceedings of the 2019 CHI Conference on Human Factors in Computing Systems*, CHI ‘19, 1–12, 10.1145/3290605.3300870 (Association for Computing Machinery, New York, NY, USA, 2019).

[44] Julesz, B. & Bergen, J. R. Human factors and behavioral science: Textons, the fundamental elements in preattentive vision and perception of textures. *The Bell System Technical Journal* **62**, 1619–1645, 10.1002/j.1538-7305.1983.tb03502.x (1983).

[45] Nagy, A. L. & Sanchez, R. R. Critical color differences determined with a visual search task. *J. Opt. Soc. Am. A* **7**, 1209–1217, 10.1364/JOSAA.7.001209 (1990). 2370588 10.1364/josaa.7.001209

[46] Treisman, A. M. & Gelade, G. A feature-integration theory of attention. *Cognitive Psychology* **12**, 97–136, 10.1016/0010-0285(80)90005-5 (1980). 7351125 10.1016/0010-0285(80)90005-5

[47] Villanueva, A. *et al*. A geometric approach to remote eye tracking. *Universal Access in the Information Society* **8**, 241–257, 10.1007/s10209-009-0149-0 (2009).

[48] Nakayama, K. & Silverman, G. H. Serial and parallel processing of visual feature conjunctions. *Nature* **320**, 264–265 (1986). 3960106 10.1038/320264a0

[49] Jeon, J., Noh, Y.-G., Kim, J. & Hong, J.-H. Pre-attentivegaze: Gaze-based authentication dataset with momentary visual interactions. *figshare*10.6084/m9.figshare.28001453 (2024). 10.1038/s41597-025-04538-3 PMC11825865 39948380

[50] Housholder, A., Reaban, J., Peregrino, A., Votta, G. & Mohd, T. K. Evaluating accuracy of the tobii eye tracker 5. In *International Conference on Intelligent Human Computer Interaction*, 379–390 (Springer, 2021).

[51] Darwish, A. & Pasquier, M. Biometric identification using the dynamic features of the eyes. In *2013 IEEE Sixth International Conference on Biometrics: Theory, Applications and Systems (BTAS)*, 1–6, 10.1109/BTAS.2013.6712724 (2013).

[52] Komogortsev, O. V., Karpov, A., Holland, C. D. & Proença, H. P. Multimodal ocular biometrics approach: A feasibility study. In *2012 IEEE Fifth International Conference on Biometrics: Theory, Applications and Systems (BTAS)*, 209–216, 10.1109/BTAS.2012.6374579 (2012).

[53] Aracena, C., Basterrech, S., Snáel, V. & Velásquez, J. Neural networks for emotion recognition based on eye tracking data. In *2015 IEEE International Conference on Systems, Man, and Cybernetics*, 2632–2637, 10.1109/SMC.2015.460 (2015).

[54] Jayawardana, Y., Jayawardena, G., Duchowski, A. T. & Jayarathna, S. Metadata-driven eye tracking for real-time applications. In *Proceedings of the 21st ACM Symposium on Document Engineering*, DocEng ‘21, 10.1145/3469096.3474935 (Association for Computing Machinery, New York, NY, USA, 2021).

[55] Alexander, R. G. & Martinez-Conde, S. *Fixational Eye Movements*, 73–115 (Springer International Publishing, Cham, 2019).

[56] Keller, E. L., Lee, B.-T. & Lee, K.-M. Chapter 2.4 - frontal eye field signals that may trigger the brainstem saccade generator. In Kennard, C. & Leigh, R. J. (eds.) *Using Eye Movements as an Experimental Probe of Brain Function*, vol. 171 of *Progress in Brain Research*, 107–114, 10.1016/S0079-6123(08)00614-6 (Elsevier, 2008). 10.1016/S0079-6123(08)00614-6 18718288

[57] Nakagawa, S., Wang, L. & Ohtsuka, S. Speaker identification and verification by combining mfcc and phase information. *IEEE Transactions on Audio, Speech, and Language Processing* **20**, 1085–1095, 10.1109/TASL.2011.2172422 (2012).

[58] Viglione, A., Mazziotti, R. & Pizzorusso, T. From pupil to the brain: New insights for studying cortical plasticity through pupillometry. *Frontiers in Neural Circuits* **17**, 10.3389/fncir.2023.1151847 (2023). 10.3389/fncir.2023.1151847 PMC10102476 37063384

[59] Pan, J., Klimova, M., McGuire, J. & Ling, S. Investigating the interaction between affective arousal and luminance in modulating pupil size. *Journal of Vision* **23**, 5414–5414 (2023).

[60] Lloyd, B., de Voogd, L. D., Mäki-Marttunen, V. & Nieuwenhuis, S. Pupil size reflects activation of subcortical ascending arousal system nuclei during rest. *ELife* **12**, e84822 (2023). 37367220 10.7554/eLife.84822 PMC10299825

[61] Kim, J. H., Yin, T., Merriam, E. P. & Roth, Z. N. Pupil size is sensitive to stimulus features independent of effects of arousal. *Journal of Vision* **22**, 4099–4099 (2022).

[62] Mukhopadhyay, S. & Nandi, S. Lpitrack: Eye movement pattern recognition algorithm and application to biometric identification. *Machine Learning* **107**, 313–331, 10.1007/s10994-017-5649-1 (2018).

[63] Harezlak, K., Blasiak, M. & Kasprowski, P. Biometric identification based on eye movement dynamic features. *Sensors* **21**, 10.3390/s21186020 (2021). 10.3390/s21186020 PMC8468647 34577223

[64] Saeed, U. Eye movements during scene understanding for biometric identification. *Pattern Recognition Letters* **82**, 190–195, 10.1016/j.patrec.2015.06.019 (2016). An insight on eye biometrics.

[65] Pfeuffer, K. *et al*. Behavioural biometrics in vr: Identifying people from body motion and relations in virtual reality. In *Proceedings of the 2019 CHI Conference on Human Factors in Computing Systems*, CHI ‘19, 1–12, 10.1145/3290605.3300340 (Association for Computing Machinery, New York, NY, USA, 2019).

[66] Castelhano, M. S., Mack, M. L. & Henderson, J. M. Viewing task influences eye movement control during active scene perception. *Journal of Vision* **9**, 6–6, 10.1167/9.3.6 (2009). 19757945 10.1167/9.3.6

[67] Pfleging, B., Fekety, D. K., Schmidt, A. & Kun, A. L. A model relating pupil diameter to mental workload and lighting conditions. In *Proceedings of the 2016 CHI Conference on Human Factors in Computing Systems*, CHI ‘16, 5776–5788, 10.1145/2858036.2858117 (Association for Computing Machinery, New York, NY, USA, 2016).

[68] Braga, R. M., Fu, R. Z., Seemungal, B. M., Wise, R. J. S. & Leech, R. Eye movements during auditory attention predict individual differences in dorsal attention network activity. *Frontiers in Human Neuroscience* **10**, 10.3389/fnhum.2016.00164 (2016). 10.3389/fnhum.2016.00164 PMC4860869 27242465

[69] Doyle, M. C. & Snowden, R. J. Identification of visual stimuli is improved by accompanying auditory stimuli: The role of eye movements and sound location. *Perception* **30**, 795–810, 10.1068/p3126 (2001). PMID: 11515953. 11515953 10.1068/p3126

[70] Hochreiter, S. & Schmidhuber, J. Long short-term memory. *Neural Computation* **9**, 1735–1780, 10.1162/neco.1997.9.8.1735 (1997). 9377276 10.1162/neco.1997.9.8.1735

[71] Lecun, Y., Bottou, L., Bengio, Y. & Haffner, P. Gradient-based learning applied to document recognition. *Proceedings of the IEEE* **86**, 2278–2324, 10.1109/5.726791 (1998).

