# Supplementary material for: Pre-AttentiveGaze: gaze-based authentication dataset with momentary visual interactions
Source: Sci Data. 2025 Feb 13;12:263. doi: 10.1038/s41597-025-04538-3 (PMC11825865; doi:10.1038/s41597-025-04538-3)
Supplement: Supplementary file 1 — Supplementary Information [file 41597_2025_4538_MOESM1_ESM.pdf]

Appendix

Stimuli design principle with formulas

Based on the designed stimuli, number of visual component (n_{VC}) is four, and number of level (n_l) except for background is also four. Variables that change in some cases are the number of targets in stimuli (t) and degree (d), which is the number of levels of each target.

Single visual component stimuli

SingleVC stimuli consist of a single visual component with 16 elements and t ranges from 1 to 4. There are two cases of configure stimuli: levels of each target singular as Figure A1(i) and duplicated as Figure A1(ii).

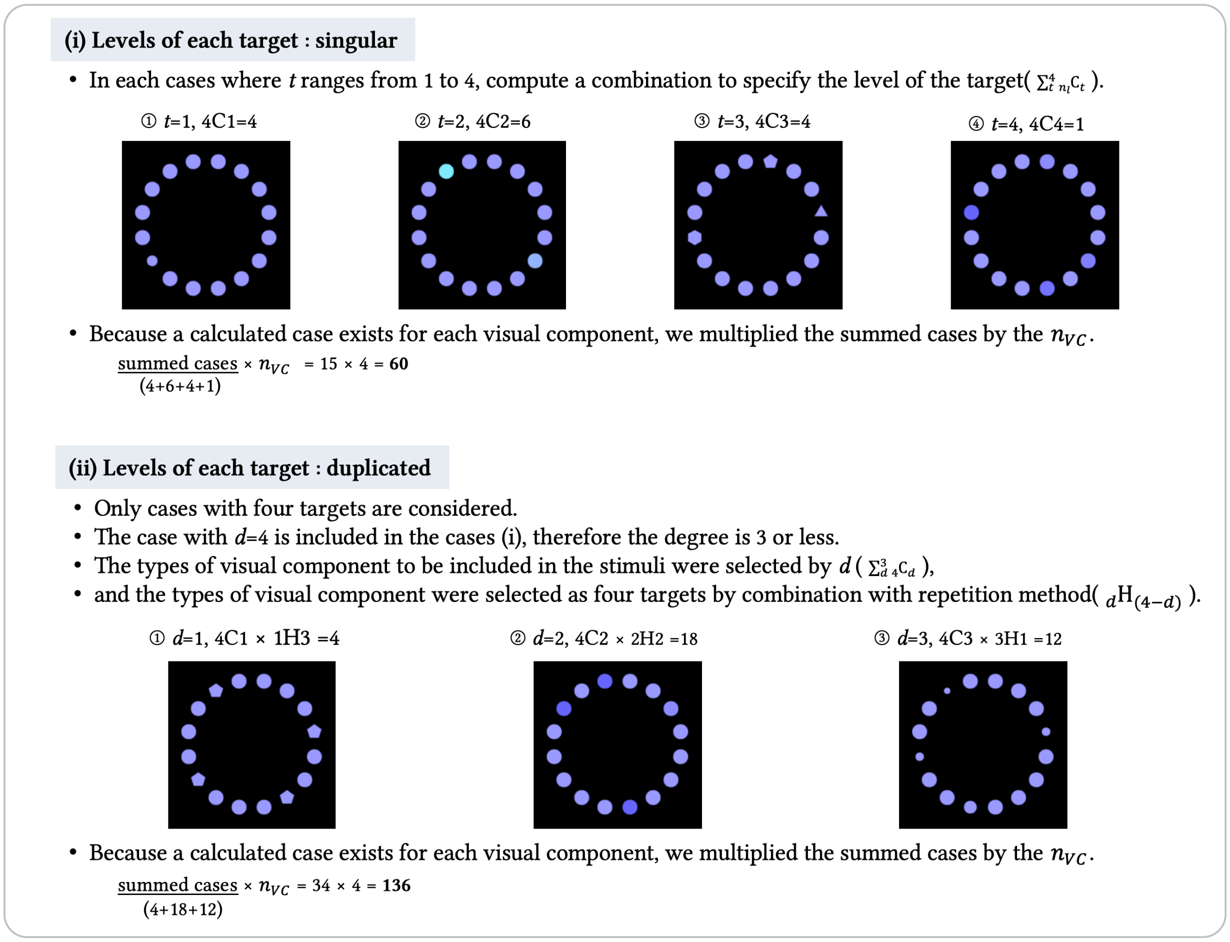

Figure A1: Example of SingleVC stimuli image according to the design case

As two cases, Figure A1(i) and (ii), are mutually exclusive events, the addition of two cases together completes formula (4).

$$SingleVC = n_{VC} \times \sum_{t}^{4} {}_{n_l}\mathrm{C}_t + n_{VC} \times \sum_{d}^{3} {}_4\mathrm{C}_d \times {}_d\mathrm{H}_{(4-d)} \tag{4}$$

Multiple visual component stimuli

MultipleVC stimuli consist of multiple visual component types. Each of the four types of visual component is selected as a target, so the number of targets t is also four. Each distractor can have a level of 1 to 4. Figure A2 shows examples of the MultipleVC stimuli. Considering the number of cases where one of the four levels is selected by the number of visual components, the following formula (5) is derived.

$$MultipleVC = ({}_{n_l}\mathrm{C}_1)^{n_{VC}} \tag{5}$$

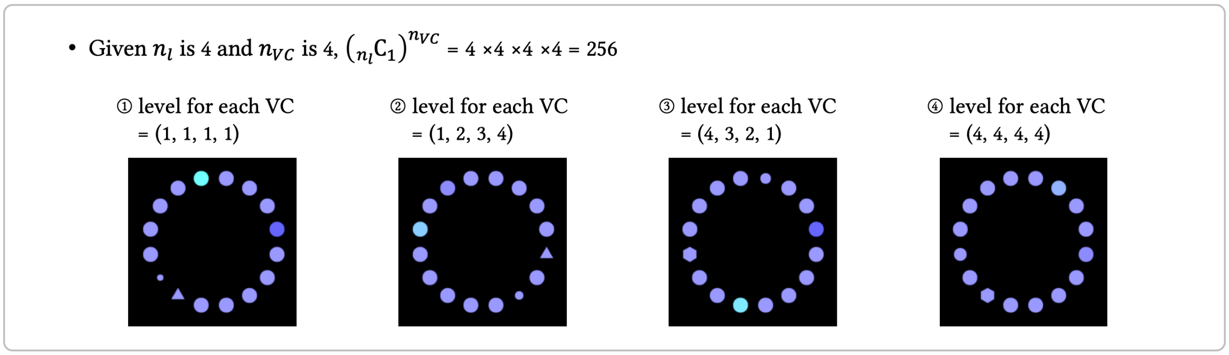

Figure A2: Example of MultipleVC stimuli image according to the design case

Demographic information about participants

ID	Gender	Age	Vision correction	Corrected VA of left eye	Corrected VA of right eye
1	M	24	non-wearers	1.0	1.0
2	M	19	contacts	0.8	0.8
3	F	22	glasses	1.0	1.0
4	M	19	non-wearers	1.0	1.0
5	F	21	glasses	1.0	1.0
6	M	19	glasses	1.0	1.0
7	F	19	non-wearers	0.9	0.9
8	M	20	non-wearers	0.5	1.0
9	F	21	non-wearers	0.8	0.8
10	F	33	contacts	1.0	0.9
11	F	20	non-wearers	1.0	1.0
12	F	19	contacts	1.0	1.0
13	F	20	glasses	1.0	1.0
14	F	23	non-wearers	1.0	1.0
15	F	23	glasses	1.0	1.2
16	F	24	non-wearers	1.0	0.9
17	M	23	non-wearers	0.8	1.0
18	M	23	glasses	0.9	0.9
19	M	26	non-wearers	1.0	1.0
20	F	32	non-wearers	0.3	0.4
21	M	33	glasses	1.2	1.2
22	M	26	non-wearers	0.7	0.7
23	F	27	non-wearers	1.0	1.0
24	F	24	non-wearers	1.0	1.0
25	M	18	glasses	1.0	1.0
26	M	26	non-wearers	0.6	0.8
27	M	19	non-wearers	1.0	1.2
28	M	19	non-wearers	1.0	0.4
29	M	20	glasses	0.8	1.1
30	M	20	glasses	0.9	1.0
31	M	17	glasses	0.9	1.1
32	M	28	glasses	1.0	1.0
33	M	19	non-wearers	1.0	1.0
34	M	26	glasses	1.0	0.8

Table A1: Demographic information about participants in the Pre-AttentiveGaze dataset abbreviation: Visual Acuity (VA)

Classification results excluding gaze features related to the pupil

This classification results were also derived using the leave-one-session-out cross-validation method. Pupil-related gaze features, such as Left pupil diameter, Right pupil diameter, and Filtered pupil diameter, are excluded during train and test dataset organization process. With the exception of the pupil-related gaze feature process, all processes were performed in the same manner as in Table 5.

**Model**	**MultipleVC**				**SingleVC**			
	**Accuracy**	**Precision**	**Recall**	**F1**	**Accuracy**	**Precision**	**Recall**	**F1**
ZeroR	.034 (.001)	.001 (.000)	.034 (.001)	.002 (.000)	.033 (.000)	.001 (.000)	.033 (.000)	.002 (.000)
DT	.294 (.054)	.297 (.048)	.294 (.054)	.292 (.052)	.286 (.043)	.293 (.041)	.286 (.043)	.286 (.042)
KNN	.298 (.062)	.334 (.060)	.298 (.062)	.290 (.059)	.286 (.045)	.327 (.048)	.286 (.045)	.279 (.044)
NB	.336 (.055)	.351 (.059)	.336 (.055)	.313 (.050)	.325 (.047)	.350 (.064)	.325 (.047)	.307 (.048)
SVM	.516 (.089)	.524 (.082)	.516 (.089)	.513 (.087)	.487 (.076)	.491 (.074)	.487 (.076)	.481 (.075)
LR	.458 (.073)	.458 (.069)	.458 (.073)	.453 (.071)	.455 (.062)	.456 (.065)	.455 (.062)	.451 (.064)
RF	.505 (.101)	.503 (.098)	.505 (.101)	.493 (.100)	.489 (.081)	.484 (.079)	.489 (.081)	.473 (.081)

Table A2: Mean (standard deviation) of classification results using leave-one-session-out cross-validation for excluding gaze features related to the pupil
